# Deconstruction of Human Age‐Related Cataract Capsules Defines Aging

**DOI:** 10.1002/advs.76532

**Published:** 2026-08-05

**Authors:** Qiaomei Tang, Ziyang Tong, Chunmei Fan, Silong Chen, Jiarui Guo, Jianghua Hu, Ke Yao, Zi Yin, Xiao Chen, Yibo Yu

**Affiliations:** ^1^ Eye Center School of Medicine The Second Affiliated Hospital Zhejiang Provincial Key Lab of Ophthalmology Zhejiang Provincial Clinical Research Center for Eye Diseases Zhejiang Provincial Engineering Institute on Eye Diseases Zhejiang University Hangzhou China; ^2^ Dr. Li Dak Sum & Yip Yio Chin Center for Stem Cells and Regenerative Medicine Center For Stem Cell and Tissue Engineering School of Medicine Zhejiang University Hangzhou China; ^3^ Department of Sports Medicine & Orthopedic Surgery The Second Affiliated Hospital and Liangzhu Laboratory School of Medicine Zhejiang University Hangzhou China; ^4^ Key Laboratory of Novel Targets and Drug Study for Neural Repair of Zhejiang Province Department of Clinical Medicine School of Medicine Hangzhou City University Hangzhou China; ^5^ Department of Ophthalmology Jiande Branch The Second Affiliated Hospital School of Medicine Zhejiang University Hangzhou China

**Keywords:** age‐related cataracts, heterogeneity, neuronal axon‐like structures, single‐cell RNA sequencing

## Abstract

Age‐related cataracts (ARC), a disease associated with aging, is the leading cause of blindness worldwide. To better understand the heterogeneous pathogenesis of ARC and identify potential therapeutic targets, we generate a comprehensive atlas of age‐related cataracts at a single‐cell resolution, encompassing three disease states—mild cataract group (Mild), severe cortical cataract group (Severe_C), and severe nuclear cataract group (Severe_N)—with a total of 230 838 lens epithelial cells (LECs) derived from the lens capsules of 554 patients. We find that ARC involves seven distinct lens capsule cell types, with notable differences in cellular composition and functional states across disease severities. Unexpectedly, we discover that neuronal axon‐like structures ingrowth into the lens is associated with cataractogenesis and its progression. All three disease groups show significant enrichment of the neurotrophic SLIT‐ROBO signaling pathway. Cluster 0 exhibits high expression of the neurotrophic factor NRG1, which forms a stable ligand‐receptor axis with the ERBB3 receptor on sympathetic neurons. These findings not only reveal the heterogeneity of ARC at the levels of cellular composition and signaling pathways but also suggest that neuro‐lens interactions may play a critical role in cataract development. This provides a new perspective for understanding the pathogenesis of age‐related cataracts.

## Graphical abstract

We generate a comprehensive atlas of age‐related cataracts (ARC) at a single‐cell resolution, encompassing three disease states—mild cataract group (Mild), severe cortical cataract group (Severe_C), and severe nuclear cataract group (Severe_N). We find that ARC involves seven distinct lens capsule cell types, with notable differences in cellular composition and functional states across disease severities. Unexpectedly, we discover that neuronal axon‐like structures ingrowth into the lens is associated with cataractogenesis and its progression. These findings not only reveal the heterogeneity of ARC at the levels of cellular composition and signaling pathways but also suggest that neuro‐lens interactions may play a critical role in cataract development.

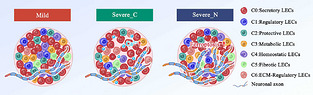

## 1 Introduction

In 2020, an estimated 94 million people were blind or visually impaired globally, and cataract is the leading cause of blindness worldwide, with age‐related cataracts (ARC) being its most prevalent form [1, 2]. This aging disease, which significantly decreases patients’ quality of life, is still one of the main ophthalmological public health problems in developed and developing countries. The global prevalence of ARC rises markedly with advancing age, from approximately 3.9% among those aged 55–64 years to over 92% in individuals aged 80 years and older. With the global population aged 60 and over projected to increase by 1.4 billion by 2030, the prevalence of ARC is expected to rise correspondingly. To date, surgical implantation of functional artificial lenses remains the only recognized effective clinical treatment method. However, there are still problems such as adverse optical phenomena after implantation, posterior capsular opacification, inflammatory reactions, and high costs, which impose a heavy burden on patients and society. Understanding how to prevent and delay the onset of cataracts holds immense social and scientific importance [2, 3].​

The pathogenesis of cataracts is complex and heterogeneous, implicating diverse molecular and cellular mechanisms. Lens epithelial cells (LECs), located beneath the anterior capsule of the lens, are the only proliferative cells within the lens and play a crucial role in maintaining lens transparency. LECs are responsible for the continuous renewal of lens fibers and protect against external damage and oxidative stress [4]. Any factor that affects the function and homeostasis of LECs can lead to cataracts. Influenced by the microenvironment of the lens capsule, LECs mediate external signals and environmental stimuli from adjacent ocular tissues, thereby controlling lens size, shape, and clarity. With aging, the function of LECs gradually deteriorates, promoting ARC development and affecting patients' visual quality [5]. To delineate the heterogeneous cellular states and functions of LECs during aging, it is imperative to identify key cellular subpopulations and regulatory networks. Disruption of any critical LEC subset can perturb lens homeostasis and drive cataractogenesis.​ Recent advances in single‐cell RNA sequencing (scRNA‐seq) now enable the comprehensive profiling of individual cells, facilitating the construction of differentiation trajectories and gene regulatory maps [6]. However, in the field of cataract research, a very limited number of studies involving single‐cell sequencing of the lens have been conducted [6, 7, 8]. There is a lack of comprehensive cell atlases of the lens capsule and studies related to disease‐associated subpopulations. Unraveling the core factors and mechanisms that govern aging remains a major goal in cataract research.

In this study, we collected LECs from 554 patients with three types of age‐related cataracts. These three types of ARC are very common in outpatient clinics. We discovered that neuronal axon‐like structures ingrowth into the lens is associated with cataractogenesis. Cluster 0, as a key subpopulation, played a vital role in this progress. The atlas revealed distinct pathogenic mechanisms underlying different states of ARC. These analyses may reveal new insights into age‐related cataract pathology and heterogeneity that could inform novel targeted treatments.

## 2 Results

### 2.1 Capsular Cell Atlas in Patients With Age‐Related Cataracts

**FIGURE 1:**
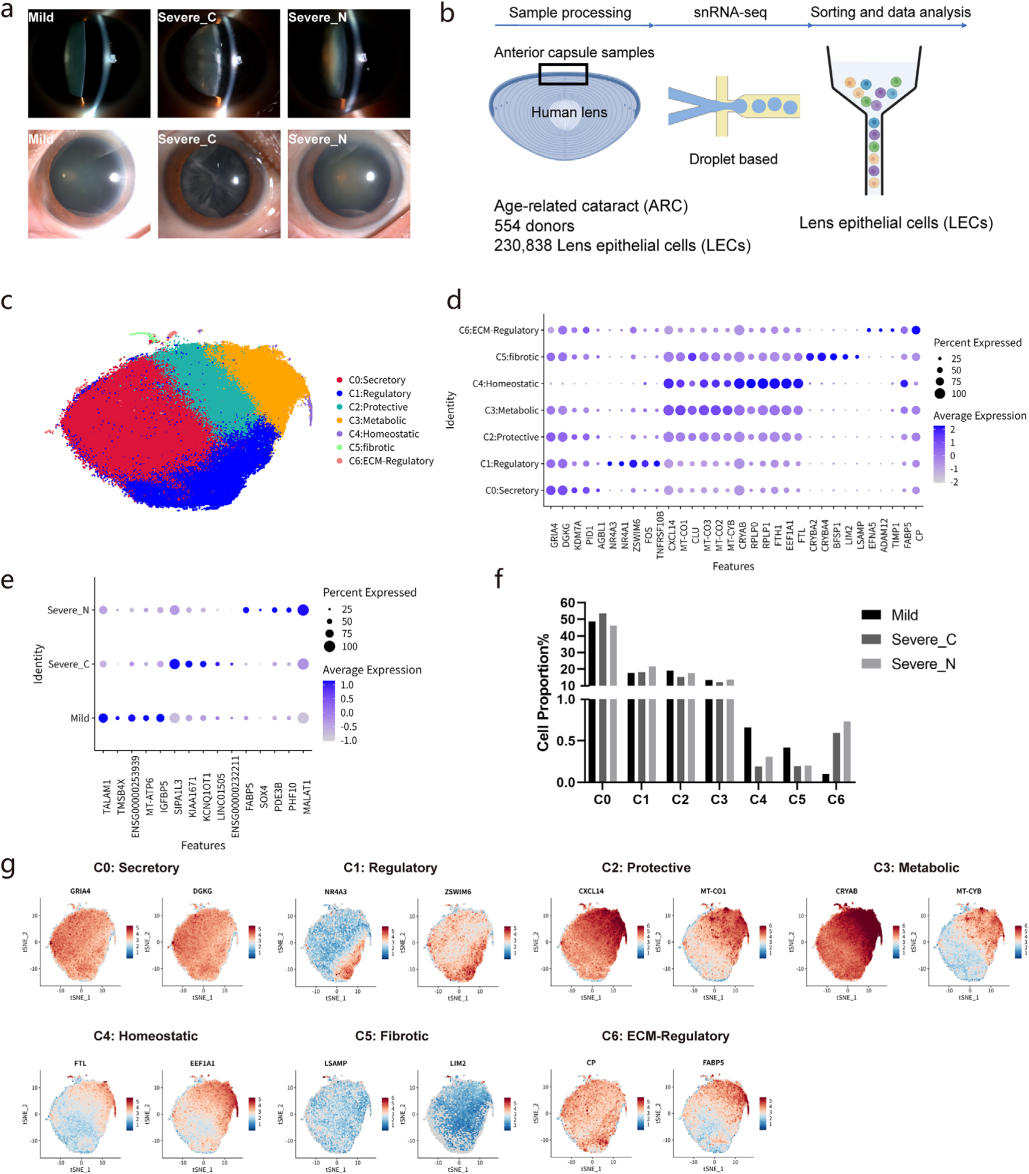
Capsular Cell Atlas in Patients with Age‐Related Cataracts. (a) Representative slit‐lamp examination images of patients with mild ARC (Mild), severe cortical ARC (Severe_C), and severe nuclear ARC (Severe_N); (b) Schematic of the data generation, study design, and integration of the atlas. (c) tSNE visualization of cell clusters from single‐nucleus RNA sequencing (snRNA‐seq) of the lens capsules; (d) Dot plot depicting expression levels of differentially expressed genes (DEGs) across lens capsule cell subpopulations; (e) Dot plot depicting expression levels of differentially expressed genes (DEGs) across cataract severity (mild ARC, severe cortical ARC, severe nuclear ARC); (f) Comparison of cell type proportions among different groups (the submitted sample is a mixed sample, this bar chart represents the total number of cells for each sample within this group); (g) Feature plots visualizing expression patterns of DEGs across lens capsule cell subpopulations.

**FIGURE 2:**
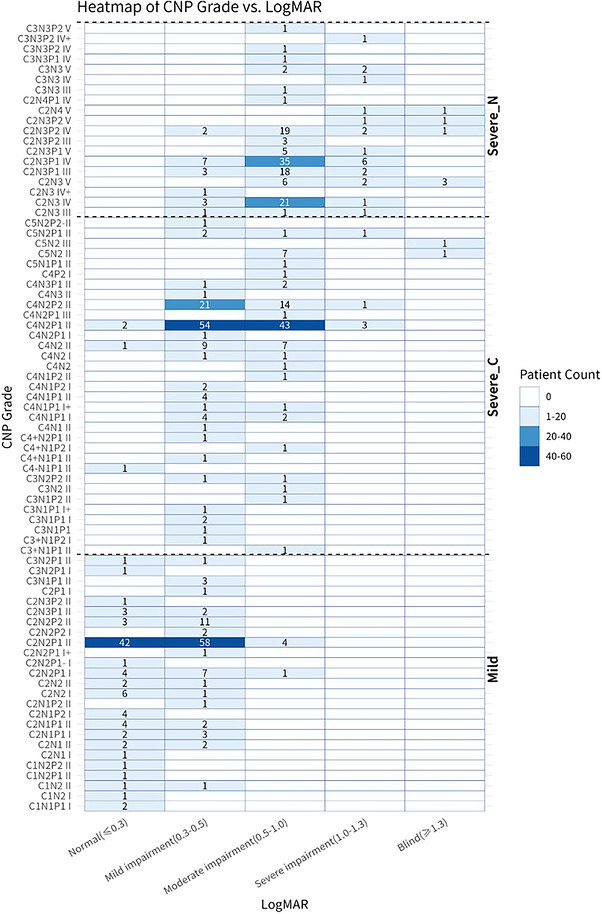
Correspondence table between cataract severity (LOCS II) and visual acuity in patients. Each number represents the number of patients.

To comprehensively understand the cellular atlas of patients with age‐related cataracts (ARC), we included 554 patient samples in this study, covering three disease stages. Patients were categorized into three groups by the same clinical doctor (Prof. Yibo Yu) based on the internationally recognized Lens Opacities Classification System II (LOCS II) [9] clinical grading standard: the mild cataract group (Mild, *n* = 185), the severe cortical cataract group (Severe_C, *n* = 210), and the severe nuclear cataract group (Severe_N, *n* = 159). Representative slit‐lamp examination images for patients in the three groups are shown in Figure 1a. As cataracts progressed from mild to severe forms (both cortical and nuclear subtypes), patients exhibited increased lens opacity with a corresponding decline in visual acuity. We also collected best‐corrected visual acuity (BCVA) data for each patient (Figure 2).

We performed single‐nucleus RNA sequencing (snRNA‐seq) on the anterior lens capsules collected from patients with age‐related cataracts (ARC), generating transcriptomic data for 230 838 lens epithelial cells (LECs). Subsequently, we systematically constructed a cellular atlas of ARC, identifying seven functionally distinct subpopulations within the aging lens capsule microenvironment. Based on their gene expression profiles, these subpopulations were designated as follows: C0: Secretory LECs, C1: Regulatory LECs, C2: Protective LECs, C3: Metabolic LECs, C4: Homeostatic LECs, C5: Fibrotic LECs, C6: ECM‐Regulatory LECs (Figure 1b–d). The Secretory LEC subpopulation (C0) exhibited a significant neural signaling association, characterized by high expression of the glutamate receptor subunit GRIA4 and signaling regulator PID1, suggesting potential involvement in neurotransmitter regulation. The Regulatory LEC subpopulation (C1) showed high expression of NR4A3 and FOS, which are implicated in immune modulation, cellular responses, and transcriptional activation. The Protective LEC subpopulation (C2) highly expressed genes involved in cytoprotection and stress responses. The Metabolic Support LEC subpopulation (C3) maintained redox balance through CRYAB and mitochondrial gene MT‐CYB. The Homeostatic LEC subpopulation (C4) expressed ribosomal proteins. The Fibrotic LEC subpopulation (C5) expressed classical lens fiber cell markers. The ECM‐Maintenance LEC subpopulation (C6) highly expressed genes involved in extracellular matrix (ECM) maintenance and remodeling (Figure 1e,g). Subsequent analysis of cellular composition proportions revealed that as the disease progressed, the proportions of subpopulations C2, C4, and C5 gradually decreased, while the proportion of C6 increased. This suggests that these subpopulations may play roles in the transition between the three disease states (Figure 1f).

### 2.2 Pathway Heterogeneity in the Lens Capsule of Patients With Age‐Related Cataracts

**FIGURE 3:**
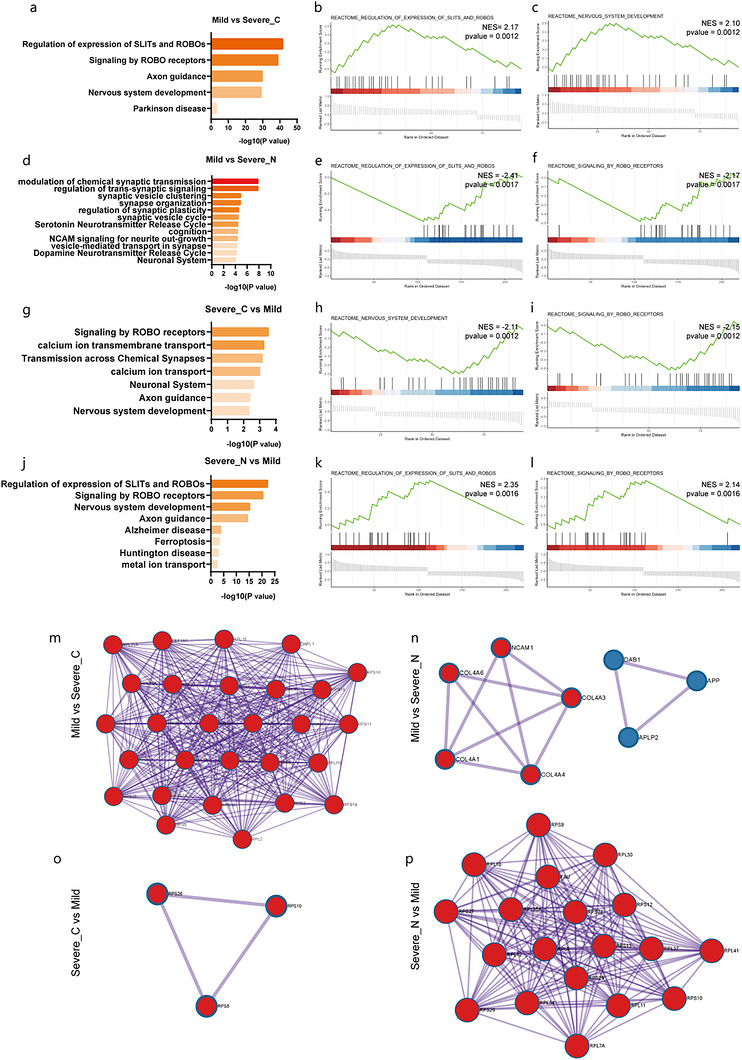
Pathway Heterogeneity in the Lens Capsule of Patients with Age‐Related Cataracts. (a) GO enrichment analysis results for upregulated differentially expressed genes (DEGs) in the Mild group compared to the Severe_C group; (b, c) GSEA enrichment analysis results for upregulated DEGs in the Mild group compared to the Severe_C group; (d) GO enrichment analysis results for upregulated DEGs in the Mild group compared to the Severe_N group; (e, f) GSEA enrichment analysis results for upregulated DEGs in the Mild group compared to the Severe_N group; (g) GO enrichment analysis results for upregulated DEGs in the Severe_C group compared to the Mild group; (h, i) GSEA enrichment analysis results for upregulated DEGs in the Severe_C group compared to the Mild group; (j) GO enrichment analysis results for upregulated DEGs in the Severe_N group compared to the Mild group; (k, l) GSEA enrichment analysis results for upregulated DEGs in the Severe_N group compared to the Mild group; (m) PPI network analysis results for upregulated DEGs in the Mild group compared to the Severe_C group; (n) PPI network analysis results for upregulated DEGs in the Mild group compared to the Severe_N group; (o) PPI network analysis results for upregulated DEGs in the Severe_C group compared to the Mild group; (p) PPI network analysis results for upregulated DEGs in the Severe_N group compared to the Mild group.

To further investigate the key signaling pathways driving the progression of age‐related cataracts (ARC), we performed gene enrichment analysis. Based on the differentially expressed genes among the various groups, Gene Ontology (GO) analysis revealed significant enrichment of genes associated with the neurotrophic SLIT‐ROBO pathway across all groups, indicating that neurotrophic signaling‐related cells are in a relatively active state during the onset and progression of ARC (Figure 3a,d,g,j). The signaling by SLIT‐ROBO modulates axonal guidance and neural progenitor proliferation [10]. The Gene Set Enrichment Analysis (GSEA) revealed that in the comparison between the Mild group and the Severe_C group, neurotrophic pathways and nervous system development pathways were upregulated in the Mild group, while the SLIT‐ROBO neurotrophic pathway was downregulated in the Severe_C group (Figure 3b,c,h,i). In the comparison between the Mild group and the Severe_N group, neurotrophic pathways and nervous system development pathways were downregulated in the Mild group, while the SLIT‐ROBO neurotrophic pathway was upregulated in the Severe_N group (Figure 3e,f,k,l). These findings indicated that neurotrophic growth‐related pathways were activated in both the Severe_N and Mild groups, whereas they were comparatively less active in the Severe_C group. Further, in comparison with the Mild group, the Severe_C group was enriched in calcium ion transport‐related pathways, and the Severe_N group was enriched in ferroptosis‐related pathways (Figure 3a,d,g,j).

Subsequently, we employed the MCODE algorithm to identify highly interconnected modules within the protein‐protein interaction (PPI) network of the ARC lens capsules. Analysis revealed that compared to the Severe_C group, the Mild group exhibited enriched protein functional modules primarily composed of large ribosomal subunit proteins, such as those from the RPL family, associated with ribosomal assembly and translation functions (Figure 3m,o). Compared to Severe_N group, the Mild group showed enrichment in two distinct functional modules: Module 1 contained COL4 (collagen type IV alpha chains), NCAM (neural cell adhesion molecule), linked to cell migration regulation; Module 2 contained APP (amyloid precursor protein), APLP2 (amyloid precursor‐like protein 2), and DAB1 (Disabled homolog 1), associated with synaptic regulation and neurodegenerative diseases (Figure 3n,p). Assessment of enrichment scores for the ribosomal protein interaction module indicated that the protein translational functional module was most active in the Severe_N group, followed by the Mild group, and least active in the Severe_C group. These findings indicated that neural pathway‐related signals may play a key role in driving the ARC.

### 2.3 Neural Ingrowth in the Age‐Related Cataract Lens Capsules

**FIGURE 4:**
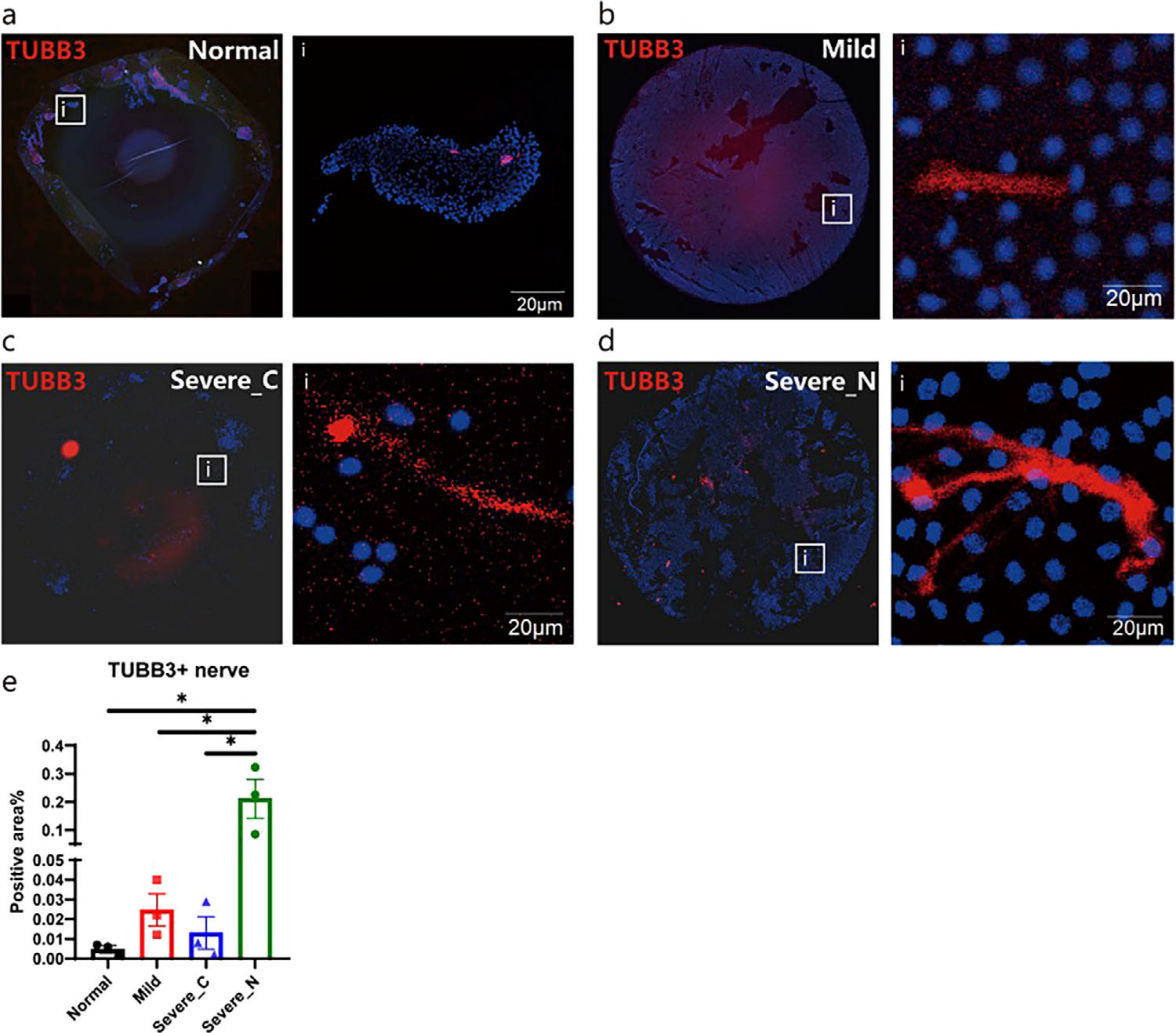
Neural Axon Staining in the Cataract Lens Capsule. (a–d) TUBB3 immunofluorescence staining of the lens capsule in Normal, Mild, Severe_C, and Severe_N groups, respectively; (e) Quantification of the TUBB3‐positive area percentage (Normal vs. Severe_N: *p* value = 0.0134; Mild vs. Severe_N: *p* value = 0.023; Severe_C vs. Severe_N: *p* value = 0.0167).

Prior findings suggest that dysregulated neural signaling may critically contribute to age‐related cataracts (ARC) progression. Therefore, we investigated the expression patterns of neural markers in ARC lesions. Immunofluorescence staining of anterior lens capsules from the ARC patients demonstrated the presence of neural axon‐like structures, as evidenced by positive labeling for the pan‐neuronal marker beta‐III tubulin (TUBB3), with axonal projections predominantly localized to the capsular periphery. Furthermore, the Severe_N group (0.2110 ± 0.06906) exhibited a higher axon‐like structure density than both the Mild group (0.02467 ± 0.008192) and the Severe_C group (0.013 ± 0.008185) (Figure 4a–d). No TUBB3‐positive axon‐like structures were detected in the epithelial cells of the transparent normal human lens capsule. However, under aging‐related stimuli, TUBB3‐positive axon‐like structures began to grow into the lens capsule tissue in all three ARC groups.

### 2.4 Microenvironment Characterization of Age‐Related Cataracts

**FIGURE 5:**
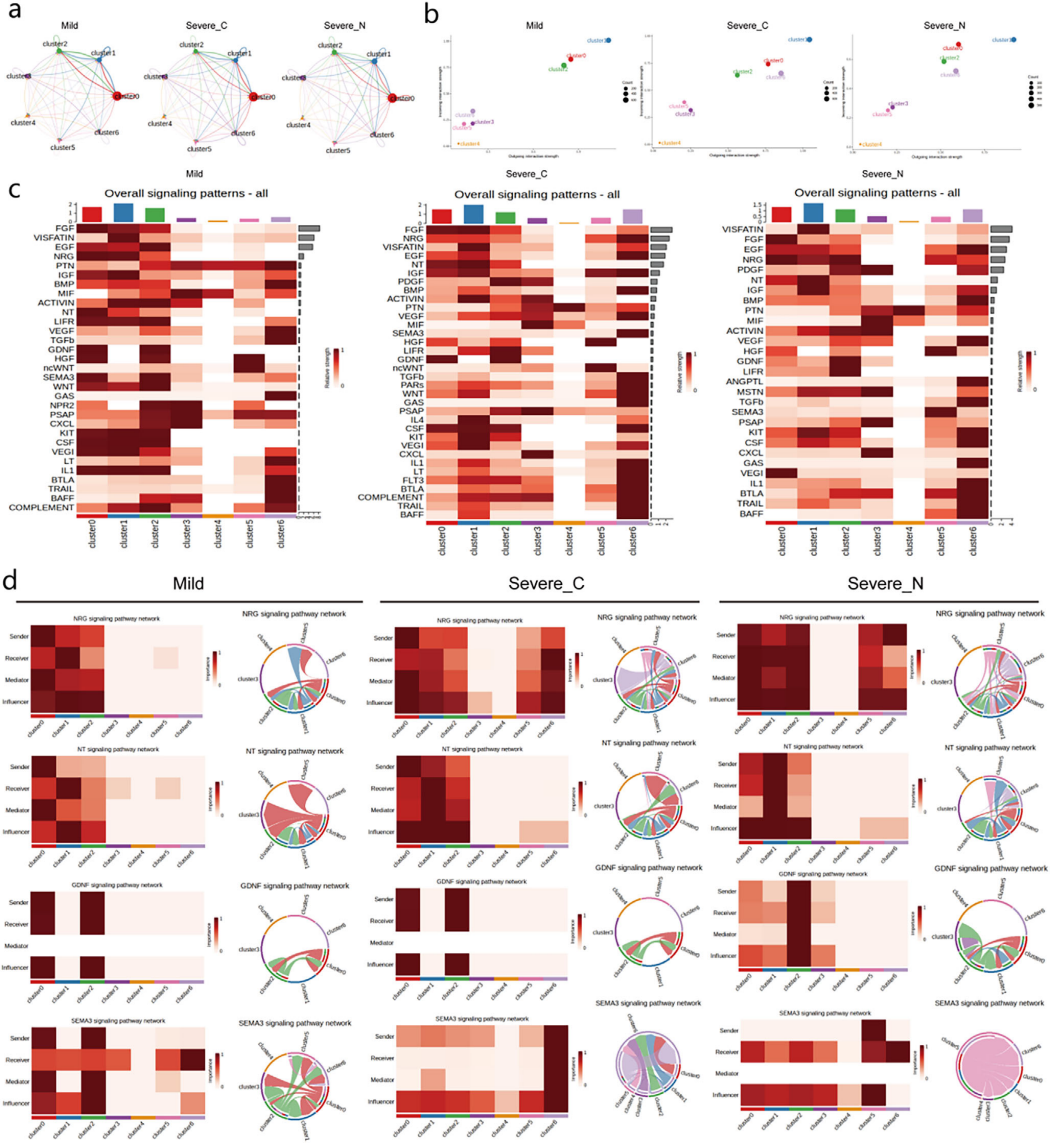
Microenvironment Characterization of Age‐Related Cataracts. (a) Circular plot depicting interaction frequencies among cell subpopulations; (b) Scatter plot of sender‐receiver subpopulations in cataract severity groups (x‐axis: outgoing communication probability, y‐axis: incoming communication probability, dot size: ligand‐receptor link count); (c) Heatmap of differentially active signaling pathways across cataract severity groups; (d) Heatmap and chord diagram of neurotrophic pathway activity patterns in cataract progression.

Intercellular communication analysis revealed that Cluster 0 exhibited the most extensive interactions with other subpopulations (Figure 5a). We visualized key signaling senders and receivers in a two‐dimensional scatter plot, with the *x*‐ and *y*‐axes representing the total outgoing and incoming communication probabilities for each cell subpopulation, respectively. Dot sizes correlate with the inferred number of ligand‐receptor links per subpopulation. The Mild group showed lower communication probabilities in Clusters 3, 4, 5, and 6, while both the Severe_C and Severe_N groups displayed greater heterogeneity in cellular interactions, with elevated communication probabilities in Clusters 3, 5, and 6. This suggests that activation of their metabolic, fibrotic, and ECM‐regulatory functions during disease progression (Figure 5b). In exploring key pathways of cell communication, we found that FGF, VISFATIN, EGF, NRG, and PTN signaling are the main subgroups of communication pathways (Figure 5c). Notably, consistent with previous findings, neural pathway‐related neuregulin (NRG) signaling was activated in the severe cataract groups. The expression pattern of SEMA3 receptors, which mediate neural growth inhibition, shifted from coordinated expression across multiple subpopulations in the Mild group to predominance in Cluster 5 regulation in the Severe_C and Severe_N groups (Figure 5c,d). This disruption of inhibitory signaling may drive abnormal axonal proliferation. Our results not only clarify molecular differences among cataract subtypes but also provide a rationale for developing targeted therapies, such as NRG1 antibodies, to modulate neurotrophic pathways.

### 2.5 Signaling Analysis of Neural Ingrowth in the Lens Capsules of ARC Patients

**FIGURE 6:**
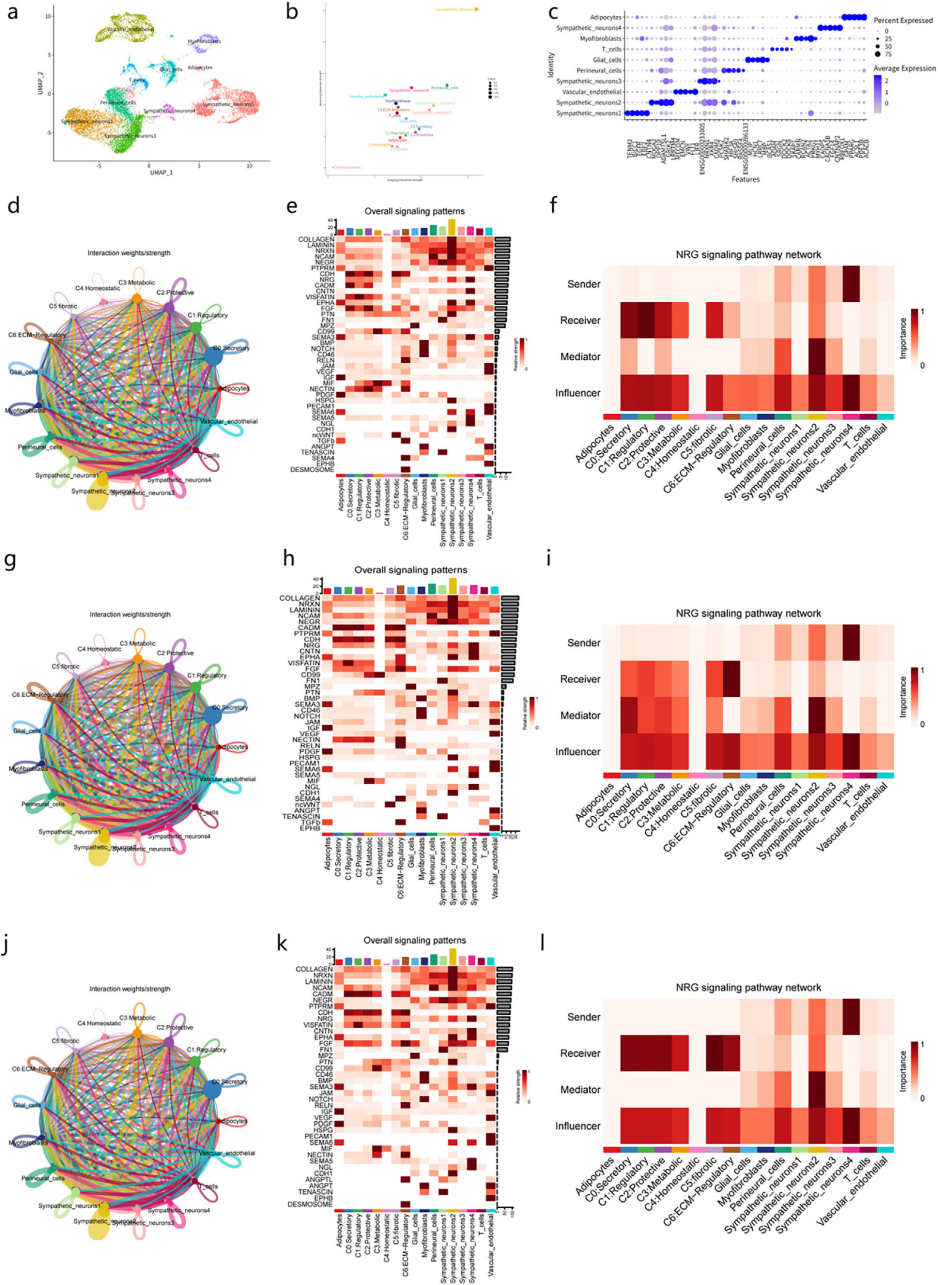
Analysis of the interaction between the capsule membrane and the sympathetic ganglion. (a) Sympathetic ganglion UMAP clustering. (b) Scatter plot of sender‐receiver subpopulations. (c) Marker molecules for subgroups of nerve cells. (d–f) The mild group and the interaction between nerve cells and key pathways were displayed. (g–i) The Severe_C group and the interaction with nerve cells and key pathways were displayed. (j–l) The Severe_N group and the interaction of nerve cells and key pathways were displayed.

**FIGURE 7:**
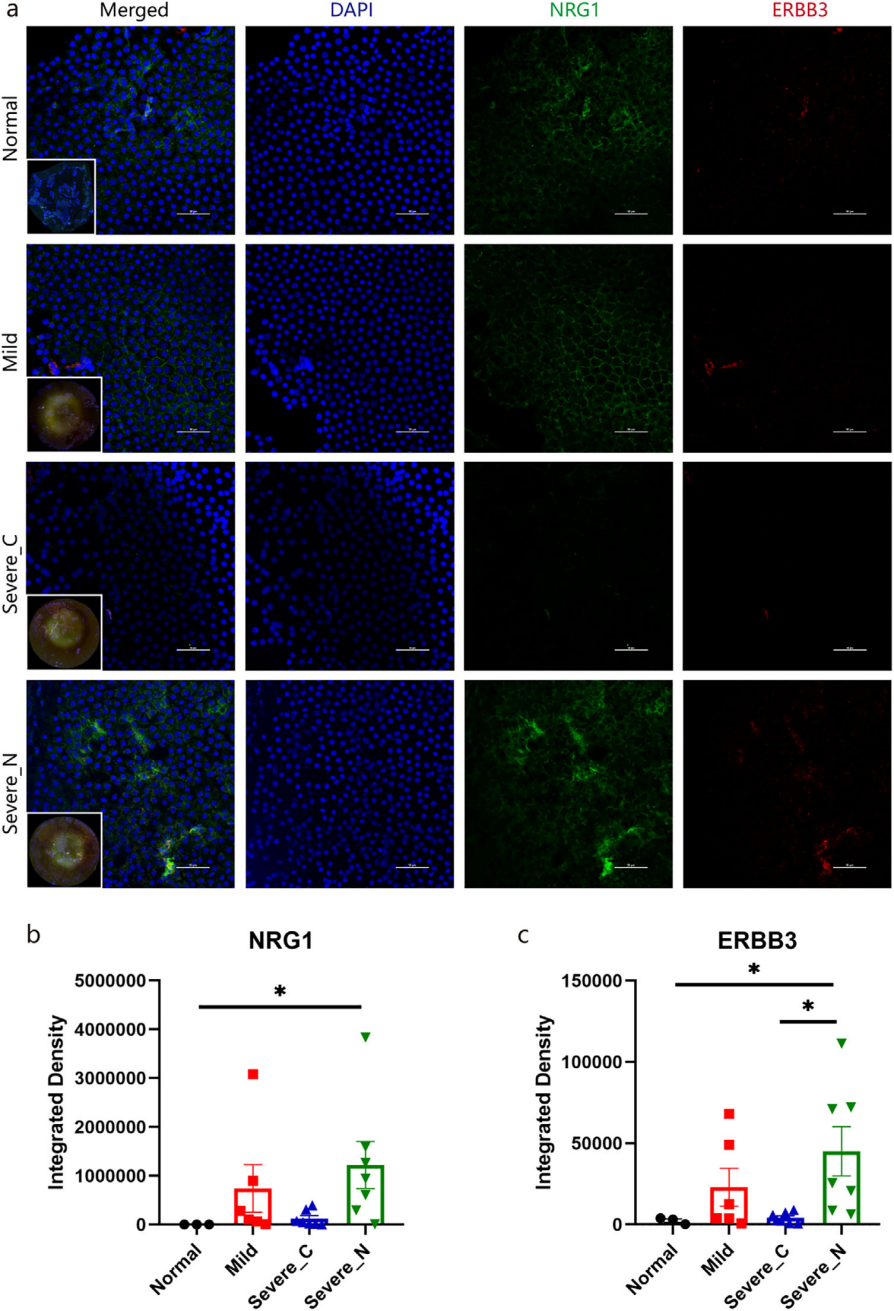
Immunofluorescence staining of NRG1 and ERBB3. (a) The results of immunofluorescence staining (Blue: nuclear, green: NRG, red: ERBB3). (b) Quantification of NRG integrated density. (Normal vs. Severe_N: *p* value = 0.0167). (c) Quantification of ERBB3 integrated density. (Normal vs. Severe_N: *p* value = 0.0461; Severe_C vs. Severe_N: *p* value = 0.0349).

To further elucidate the mechanisms of neural regulation in the pathogenesis of ARC, we performed interaction analyses between human sympathetic ganglia and lens capsule epithelial cells [11]. Single‐nucleus RNA sequencing (SnRNA‐seq) data from human sympathetic ganglia were utilized, comprising 10 distinct subclusters (Figure 6a). Among these, Sympathetic_neurons2 exhibited the strongest signaling activity in both sending and receiving, identifying it as the most interactive subpopulation (Figure 6b). Differentially expressed genes in each group were shown in Figure 6c; notably, high expression in Sympathetic_neurons2 was associated with the synaptic neurotransmitter MDGA2, tissue repair gene ADAMTSL1, and LRRTM4, a regulator of glutamatergic synapse assembly. Overall interaction patterns highlighted Cluster 0 and Sympathetic_neurons2 as the most active subpopulations (Figure 6d,g,j). Heatmaps of interaction pathways revealed that pathways involving multiple subpopulations included COLLAGEN, LAMININ, NRXN, and NCAM (Figure 6e,h,k). In severe cataracts, neurotrophic NRG pathway‐related cell–cell communication signals were intensified (Figure 6f,i,l). Key subpopulations contributing to neurotrophic signaling included cluster 0, Sympathetic_neurons2, and Sympathetic_neurons4. The core mechanism centers on the NRG1‐ERBB3 axis: cluster 0 highly expresses neurotrophic factor NRG1, forming a stable ligand‐receptor pair with ERBB3 on sympathetic neurons. Immunofluorescence staining of anterior lens capsules from ARC patients demonstrated expression of both NRG1 and ERBB3 across all groups. Compared with the normal group (3670 ± 133.6), the expression level of NRG1 in the Severe_N group (1220919 ± 481839) was significantly increased (*p‐*value = 0.0167). Furthermore, compared with both the normal group (2366 ± 1107) and Severe_C group (4111 ± 1237), the expression level of ERBB3 in the Severe_N group (45063 ± 15127) was significantly increased (Normal vs. Severe_N: *p* value = 0.0461; Severe_C vs. Severe_N: *p* value = 0.0349) (Figure 7). Together, these findings offer a potential molecular basis for neural ingrowth associated with cataract progression.

### 2.6 Secretory Lens Epithelial Cells (LECs) With Neurofunctional Propensity

**FIGURE 8:**
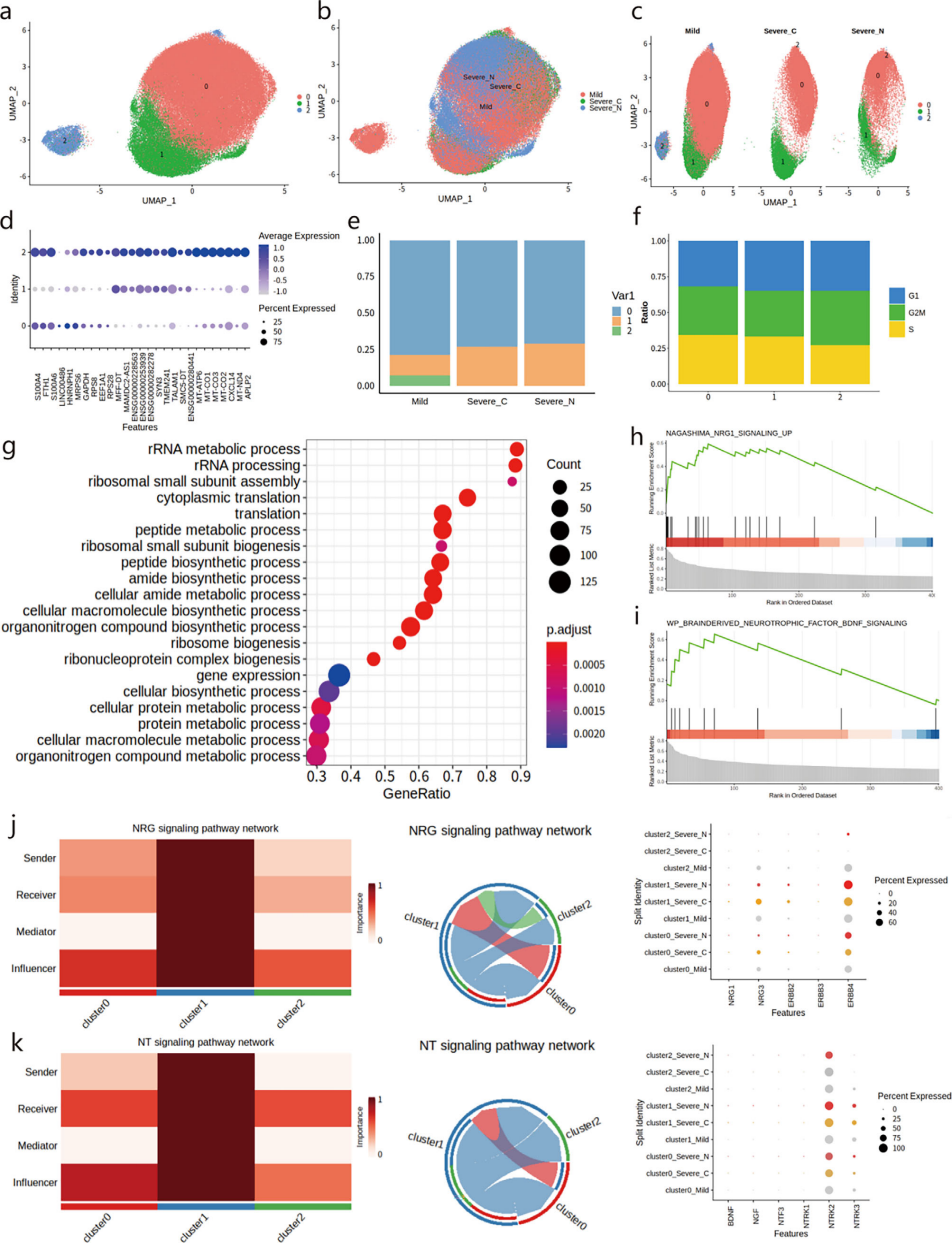
Subcluster Analysis of C0 Secretory LECs. (a) UMAP visualization of Cluster 0 subclustering; (b, c) UMAP visualization of lens capsule snRNA‐seq subclusters stratified by cataract severity (Mild, Severe_C, Severe_N) with color‐coding indicating Cluster 0 subtypes; (d) Dot plot of differentially expressed genes across Cluster 0 subpopulations; (e) Proportional composition of Cluster 0 subpopulations; (f) Cell cycle phase distribution across subpopulations; (g) Dot plot of significant GO terms enriched in subcluster C1; (h, i) GSEA enrichment plots for subcluster C1; (j, k) Heatmap and chord diagram depicting neurotrophic pathway activity.

Integrated analysis of neuron‐microenvironment interactions identified Cluster 0 as the central functional subpopulation in age‐related cataracts (ARC). This cluster was further subdivided into three subpopulations:C0 – S100A4+ LECs, C1 – SYN3+ LECs, and C2 – MT‐CO1+ LECs (Figure 8a–d). During disease progression, we observed expansion of C1, characterized by upregulated neurotrophic signaling, and depletion of C2, which was associated with mitochondrial dysfunction (Figure 8e). Pathway enrichment analysis revealed elevated neurotrophic pathway activity, including NRG and neurotrophin (NT) ligand‐receptor systems, predominantly in C1, especially in Severe_C and Severe_N groups. These findings established SYN3+ LECs as a pivotal neurotrophic regulatory subpopulation driving ARC pathogenesis (Figure 8g–k).

## 3 Discussion

This study focuses on the impact of aging on lens tissue, with particular emphasis on age‐related cataracts (ARC). Elucidating the pathological alterations within the lens epithelial cells during lens aging is critically important. We analyzed 23 0838 cells from 554 patients across three types of ARC. Our findings reveal that ARC comprises seven distinct lens capsule cell types, exhibiting notable differences in cellular composition and functional states corresponding to disease severity. Importantly, this study uncovers that neuronal axonal ingrowth into the lens is associated with cataract development and progression. These observations raise two key questions: What drives the abnormal proliferation of nerve axon‐like structures, and what is the origin of the nerve axon‐like structures detected within the lens capsule tissue?

The lens is an avascular and aneural tissue. Current research predominantly focuses on the critical role of lens epithelial cells (LECs) in maintaining lens transparency [12, 13, 14]. No nerve axon‐like structures have been detected within the epithelial cells of the transparent human lens capsule. However, under aging stimuli, nerve axon‐like structures begin to grow into the lens capsule tissue. Upon initiation of neurotrophic signaling, the intensity of these signals varies with the severity and state of age‐related cataracts as the disease progresses. The SLIT‐ROBO neurotrophic pathway was upregulated in both Severe_N and Mild groups, while being comparatively less active in the Severe_C group. This pattern was consistent with the TUBB3 staining results of anterior lens capsules from ARC patients. Previous studies have identified senescent Schwann cells as significant barriers to axonal regeneration in aging and chronic denervation, secreting inhibitory factors that directly impair axon growth [15]. However, the authoritative reviews suggest that age‐related regenerative impairment is primarily due to alterations in peripheral neural pathways and target tissues rather than intrinsic neuronal growth capacity. Other research identifies that changes in neurotrophic factors (NGF, BDNF) and extracellular matrix components (laminin) contribute significantly to reduced axonal sprouting and reinnervation in aged animals [16, 17]. In our study, activation of the neurotrophic pathway was detected in the lens capsules of the ARC patients, followed by the ingrowth of nerve axon‐like structures into senescent capsules. Integrated analysis of neuron‐microenvironment interactions identified Cluster 0 as the central functional subpopulation in ARC, subdivided into S100A4+ LECs, SYN3+ LECs, and MT‐CO1+ LECs. During the progression from Mild to severe ARC (Severe_C and Severe_N groups), the proportion of SYN3+ LECs, associated with neurotrophic signals, significantly increased, whereas the proportion of MT‐CO1+ LECs, linked to mitochondrial function, significantly decreased. Previous studies report that Synapsin III (SYN3) expression decreases in mature lenses but increases in aged lenses, potentially linked to age‐related stress responses [18]. Moreover, mitochondrially encoded cytochrome c oxidase I (MT‐CO1), a subunit of the respiratory chain complex IV, plays a pivotal role in lens function and cataractogenesis. In early cataract development, MT‐CO1 transcription is upregulated to enhance ATP production and maintain cellular function; however, in advanced lens opacification, MT‐CO1 expression and ATP levels decline markedly, leading to energy‐metabolic imbalance and lens clouding [19, 20]. These findings align well with our observations in the present study.

Our findings indicated that aging stimulated the neurotrophic NRG signaling pathway, thereby promoting the ingrowth of axon‐like structures, which may have contributed to LEC abnormalities and subsequent cataract formation. Notably, Cluster 0 exhibited high expression of the neurotrophic factor NRG1, which establishes a stable ligand‐receptor axis with the ERBB3 on sympathetic neurons. It is well known that lens accommodation is primarily governed by the parasympathetic pathway through the Edinger–Westphal nucleus and the ciliary ganglion, leading to ciliary muscle contraction and zonular relaxation for near focusing. In contrast, the supplementary sympathetic pathway exerts an inhibitory, disaccommodative effect, fine‐tuning resting tension and preventing excessive accommodation. With aging, stiffening of the lens and ciliary body diminishes parasympathetic efficacy, while residual sympathetic inhibition may further exacerbate the decline in accommodation [11]. Importantly, tissue remodeling and inflammation are increasingly recognized as permissive microenvironmental cues for nerve growth and axonal ingrowth. In osteoarthritic joints, inflammatory and remodeling changes are accompanied by NGF induction and neural remodeling [21]. Likewise, in peripheral nerve repair, transiently reshaping the inflammatory microenvironment can enhance macrophage recruitment and promote axon extension, underscoring the close coupling between inflammation, matrix remodeling, and neurite outgrowth [22]. The lens capsule is the basal membrane that completely surrounds the lens and has a protective function. It can activate the inflammatory response and remodeling process of the lens epithelium after surgery or injury. Recent work [23] has further highlighted the biological relevance of ERBB‐family signaling in lens pathology. The study showed that ERBB signaling is closely associated with lens epithelial cell fibrosis, epithelial‐mesenchymal transition, and fibrotic progression after cataract surgery, supporting the notion that this pathway may actively participate in lens remodeling and disease progression. Consistent with this study, the NRG1/ErbB4 signaling pathway is known to regulate GABAergic transmission in the brain and has been implicated in several neuropsychiatric disorders. Moreover, the NRG1/ERBB3‐PI3K/Akt signaling axis has shown therapeutic potential in preserving sensory functions during aging and in neurodegenerative diseases [24]. Moreover, previous studies also demonstrate that NRG1 significantly suppresses stress‐induced premature senescence in vascular cells in vitro and in the aorta of diabetic mice in vivo. Conversely, loss of the NRG1 receptor ErbB4 induces cellular senescence both in vitro and in vivo [25]. Consistent with these observations, immunofluorescence staining of anterior lens capsules from patients with ARC revealed the presence of neural axon‐like structures, evidenced by positive labeling for the NRG1 and ERBB3. These results indicate that the NRG1/ERBB3 axis regulated by the key subpopulation Cluster 0 may be related to the neurogenic driving mechanism associated with ARC. Targeting axon‐like structure ingrowth and ErbB‐related signaling may therefore represent a promising direction for future mechanistic studies and therapeutic exploration in cataract treatment.

Intercellular communication analysis revealed a distinct shift in SEMA3 receptor expression patterns: from coordinated regulation across multiple subpopulations in the Mild group to regulation dominated by cluster 6 in the Severe_C group and by cluster 5 in the Severe_N group. This breakdown of inhibitory signaling may underlie aberrant axonal proliferation. Previous studies have shown that Sema3A forms complexes with Neuropilin (NRP) and Plexin receptor to exert axon guidance through repulsion, thereby preventing axons from invading inappropriate regions and, in some cases, inducing neuronal apoptosis [26]. Dysregulation of the SEMA3 signaling pathway during aging weakened the control of axonal polarity, leading to mislocalization and pathological axonal ingrowth [27]. Consequently, aging‐related alterations in neural NRG‐SEMA3 signaling were associated with axonal invasion into the lens capsule and with the formation of an abnormal microenvironment in age‐related cataract. Our results further identified significant enrichment of calcium channel activity‐related pathways in the Severe_C group and ferroptosis‐related pathways in the Severe_N group, compared to the Mild group. This indicated that these signals were associated with the progression of ARC. Calcium signaling and homeostasis are well established as critical factors in cataract formation. Altered calcium signaling in lens epithelial cells (LECs) accelerates cataractogenesis, as reflected by faster propagation yet reduced amplitude and prolonged duration of calcium waves, pointing to disrupted intercellular communication [28]. Elevated intracellular calcium activates calpain proteases, leading to irreversible degradation of lens structural proteins, a key mechanism underlying cataract development [29]. Consistently, animal studies demonstrate that calcium precipitates accumulate in the lens cores during age‐related nuclear cataract formation, highlighting calcium precipitation as a contributor to lens opacity [30]. In addition to calcium signaling, ferroptosis has emerged as a central mechanism in cataract pathogenesis. In ARC, LECs display the three hallmarks of ferroptosis: labile iron accumulation, elevated reactive oxygen species (ROS), and increased lipid peroxidation [31]. Melatonin has been shown to delay cataract formation by suppressing lipid peroxidation through the SIRT6/p‐Nrf2/GPX4 and SIRT6/NCOA4/FTH1 pathways, identifying novel therapeutic targets for ARC [32]. Importantly, inhibition of ferroptosis significantly attenuates highly myopic cataract progression [33]. A recent study profiled 18 596 single cells from human lens superficial tissue using scRNA‐seq and identified two distinct LEC subtypes, characterized by C8orf4 and ADAMTSL4 expression. These subtypes were proposed as lens epithelial stem/progenitor cells, providing further insights into lens biology and cataract development [6].

A limitation of this study lies in the inherent requirement of single‐cell RNA sequencing to dissociate tissue samples, thereby disrupting intercellular relationships. In contrast, spatial transcriptomic technologies bypass tissue dissociation while preserving spatial architecture, enabling gene expression to be mapped across thousands of cells within their native tissue context. Building on existing studies, we plan to employ spatial omics in future work to investigate the neuro–lens tissue architecture, quantify neuronal density, delineate lesion regions, and examine neuron–microenvironment interactions. In addition, functional validation of neuronal axonal growth in vitro or in organoid‐based models will be an important direction for future research. This approach will yield deeper insights into the contribution of neuro–lens interactions to cataract pathogenesis and represents the main focus of our subsequent research.

In conclusion, our comprehensive findings reveal the heterogeneity of age‐related cataract (ARC) at both the cellular composition and signaling pathway levels, while also highlighting the critical role of neuro–lens interactions in disease development. This study not only advances the understanding of ARC biology but also provides potential avenues for the development of targeted therapeutic strategies.

## 4 Experimental Section

### 4.1 Tissue Samples From Patients With ARC and Donors

This study was conducted at the Eye Center, Second Affiliated Hospital, School of Medicine, Zhejiang University, following approval from the institutional research and ethics committee [(2022)LSYD(0752)]. All procedures adhered to the principles of the Declaration of Helsinki. A total of 554 patients diagnosed with age‐related cataract (ARC) and undergoing cataract surgery were enrolled between October 2022 and December 2024. Informed consent for the use of clinical data and lens capsules was obtained from all participants. Normal anterior lens capsules were obtained from three healthy organ donors through the Eye Bank of the Second Affiliated Hospital of Zhejiang University. A total of six lenses were included in this study, all of which had no history of ocular disease and exhibited clear lenses under a stereomicroscope examination. Written informed consent for the use of these samples was obtained from the guardians or legally authorized representatives of the organ donors. These normal anterior lens capsules from donors were only used for Immunofluorescence validation.

### 4.2 Isolation of Anterior Lens Capsules

Circular anterior lens capsules with a diameter of 5 mm were excised during cataract surgery, collected, and stored in liquid nitrogen until use. Single‐nucleus suspensions were prepared by Genedenovo Biotechnology Co., Ltd. (Guangzhou, China). A small‐scale pooling strategy was implemented, whereby 7–10 individual samples of identical clinical grade were combined to construct one sequencing library. Briefly, capsules were transferred from cryotubes to a homogenizer, followed by the addition of citrate–phosphate lysis buffer (0.3 mol/L) for 1 min. The tissue was homogenized using a pestle, after which a nuclear wash buffer was applied and the filtrate collected. Iohexol was added to the filtrate, mixed thoroughly by pipetting, and the nuclear suspension was carefully extracted. Nuclear staining was performed for quality assessment, followed by fluorescence‐activated nuclear sorting (FANS).

### 4.3 snRNA‐seq Library Preparation and Sequencing

Nuclei were loaded onto the 10x GENOMICS GemCode single‐nucleus platform to generate single‐nucleus Gel Bead‐In‐Emulsions (GEMs). Reverse transcription and library preparation were performed using the Chromium Next GEM Single Nucleus 3′ Reagent Kit v3.1 (10x Genomics, CG000204) according to the manufacturer's instructions. Sequencing was carried out on the Illumina NovaSeq 6000 system. Library construction and sequencing were conducted by Genedenovo Biotechnology Co., Ltd (Guangzhou, China).

### 4.4 snRNA‐seq Data Preprocessing and Analysis

Raw sequencing reads were processed using Cell Ranger (version 7.1.0) with default and recommended parameters. Gene–barcode matrices were generated for each sample by counting unique molecular identifiers (UMIs) and removing non‐cell‐associated barcodes. High‐quality barcoded nuclei and gene expression counts were compiled into gene–barcode matrices. Data were subsequently imported as Seurat objects (version 4.3.0.1) in R (version 4.0.5) [34]. Low‐quality nuclei were removed using the thresholds: nFeature <200 or >6000 and mitochondrial UMI fraction >10%. Data normalization (normalizeData function, Seurat package) was applied, and highly variable genes were extracted. Batch effects were corrected, and datasets were integrated using Harmony [35].

### 4.5 Differential Gene Expression Analysis

Principal component analysis (PCA) was performed on the top 2000 variable genes, and the top 20 principal components were used for clustering analysis. t‐SNE was applied for dimensionality reduction. Clusters were identified via Seurat's FindClusters function using a K‐nearest neighbor (KNN) graph‐based model, with resolution adjusted for granularity. Differentially expressed genes (DEGs) in each cluster were identified using the FindAllMarkers function in Seurat.

### 4.6 Identification of Cell Types and Subtypes

Cell clusters were annotated using canonical marker genes. Visualization was conducted with Seurat functions including FeaturePlot and DotPlot, generating modularity heatmaps, t‐SNE plots, and dot plots of marker gene expression. Cell‐type distributions were quantified as percentages within each cluster, and scaled average gene expression per cell type was visualized with bar plots.

### 4.7 Enrichment Analysis

Gene Ontology (GO) analysis [36] was performed to determine the biological functions of DEGs in the Mild, Severe_C, and Severe_N groups using Metascape [37]. The pipeline identified statistically enriched terms (GO and KEGG) using accumulative hypergeometric p‐values and enrichment factors. Terms were clustered hierarchically into trees based on Kappa‐statistical similarities in gene memberships, with a 0.3 Kappa score threshold applied to define term clusters. Protein–protein interaction (PPI) networks for input genes were extracted and analyzed, followed by application of the MCODE algorithm to identify densely interconnected protein neighborhoods.

### 4.8 Gene Set Enrichment Analysis (GSEA)

GSEA was an unsupervised analytical approach with high sensitivity to subtle gene expression changes. The c2.all.v2024.1.Hs.entrez.gmt database was used as the reference gene set. Normalized enrichment scores (NES) were calculated, and significance was defined as P < 0.05.

### 4.9 Cell Communication Analysis

Cell–cell communication was inferred using CellChat (version 1.5.0) [38]. The CellChat package integrates a curated ligand–receptor interaction database spanning diverse cell types and signaling pathways. The analysis workflow included: identification of overexpressed genes, computation of communication probabilities, and detection of statistically significant intercellular communications.

### 4.10 Immunofluorescence

Lens capsules were fixed in 4% paraformaldehyde (PFA) for 24 h at 4°C, followed by PBS washes for 15 min (5 min × 3). Samples were then blocked in QuickBlock Blocking Buffer (Beyotime, P0220) for 1 h at room temperature. Subsequently, sections were incubated overnight at 4°C with primary antibodies: Anti‐beta III Tubulin (TUBB3, 1:500, Abcam, ab18207), NRG1 (1:200, Abcam, ab53104), and ERBB3 (1:200, Santa Cruz, sc‐415). The secondary antibodies were Alexa Fluor 488 fluorescent dyes (Thermo Fisher Scientific, 1:400) and Alexa Fluor 561 fluorescent dyes (Thermo Fisher Scientific, 1:400). The percentage of fluorescence‐positive cells was quantified from 3–5 randomly selected fields per capsule.

### 4.11 Statistical Analysis

All data were presented as mean ± SEM (standard error of the mean). Normality was assessed using the Shapiro‐Wilk test, and when p>0.05, comparisons among groups were performed using one‐way analysis of variance (ANOVA) (two‐sided) and Tukey's honestly significant difference (HSD) post hoc test for multiple comparisons without adjustment for potential covariates such as age or sex, as the primary aim of this analysis was to assess the unadjusted association with disease status. When p<0.05 for the Shapiro‐Wilk test, comparisons among groups were analyzed by non‐parametric Kruskal‐Wallis H test (two‐sided) and Dunn's post hoc test for multiple comparisons without adjustment for potential covariates. Statistical significance was defined as P < 0.05 (95% confidence interval). Graphs were generated using GraphPad Prism (version 8.0.1). The sample size of each group was represented by scatter plots, and each measurement value comes from a different sample.

## Author Contributions

Y. Y. B., C. X., Y. Z., and Y. K.: conceptualization. T. Q. M., T. Z. Y., F. C. M., C. S. L., G. J. R., and H. J. H.: methodology. YYB, T. Q. M., G. J. R., C. S. L., and H. J. H.: investigation. T. Z. Y. and F. C. M.: visualization. Y. Y. B., C. X., Y. Z., and Y. K.: Supervision. T. Q. M., T. Z. Y., and F. C. M.: writing – original draft. Y. Y. B., C. X., Y. Z., and Y. K.: writing – review & editing.

## Funding

This work was supported by the National key research and development program of China (2022YFA1106800); the National Natural Science Foundation of China (82371036, T2121004, 82222044, 32471211); the Key Research and Development Program of Zhejiang Province (2025C02156); the Key R&D Program of Zhejiang (2024SSYS0026); the Fundamental Research Funds for the Zhejiang Provincial Universities (K20240141).

## Conflicts of Interest

The authors declare no conflicts of interest.

## Data Availability

All sequencing files of lens capsules supporting the findings of this study are publicly available in the SRA database under the BioProject accession number PRJCA040144. The single‐nucleus RNA‐seq data of human sympathetic ganglia were obtained from the GSE241386 dataset. Additional information necessary to reproduce or reanalyze the results is available from the corresponding author upon reasonable request.
